# Bacteremic skin and soft tissue infection caused by *Prevotella loescheii*

**DOI:** 10.1186/1471-2334-14-162

**Published:** 2014-03-24

**Authors:** Mansoor Mehmood, Nabil A Jaffar, Muhammad Nazim, Faisal A Khasawneh

**Affiliations:** 1 Section of Infectious Diseases, Department of internal medicine, Texas Tech University Health Sciences Center, 1400 S. Coulter Street, Amarillo, TX 79106, USA; 2 Department of Surgery, Texas Tech University Health Sciences Center, Amarillo, Texas, USA

**Keywords:** Anaerobe, Bacteremia, *Prevotella loescheii*

## Abstract

**Background:**

Anaerobes are a major component of gut flora. They play an important role in the pathogenesis of infections resulting from breaches in mucus membranes. Because of the difficulties in cultivating and identifying it, their role continues to be undermined. The purpose of this paper is to report a case of *Prevotella loescheii* bacteremic skin and soft tissue infection and review the literature.

**Case presentation:**

A 42-year-old Caucasian man was admitted for an elective bariatric surgery. A lengthy intensive care unit stay and buttocks decubitus ulcers complicated his post-operative course. After being transferred to a long-term care facility, the decubitus ulcer became secondarily infected with multiple bacteria including *P. loescheii;* an anaerobe that grew in blood and wound cultures. The patient was treated successfully with aggressive surgical debridement, antibiotics and subsequent wound care.

**Conclusion:**

*P. loescheii* colonizes the gut and plays an important role in periodontal infections. In rare occasions and under suitable circumstances, it can infect skin and soft tissues as well as joints. Given the difficulties in isolating anaerobes in the microbiology lab, considering this bacterium alongside other anaerobes in infections of devitalized tissue is indicated even if cultures were reported negative.

## Background

Decubitus ulcers are a common complication of chronic critical illness [1]. The problem becomes more devastating when those wounds become secondarily infected. This infection is usually poly-microbial [2]. Culprit pathogens include streptococci, staphylococci and enterobacteriaeae [3]. Anaerobes, spreading from adjacent mucosal surfaces, can play a role in this infection especially when there is ischemia and significant tissue necrosis [2]. Bacteroides, peptostreptococcus and clostridium species are the most common anaerobic isolates [4]. Anaerobic bacteremia complicating this infection is rarely encountered.

We report a case of infected decubitus ulcer caused by multiple microorganisms including *Prevotella loescheii*. This anaerobe was also isolated in blood cultures, which has not been previously reported.

## Case presentation

A 42 year-old white male with a past medical history significant for morbid obesity (body mass index of 41) admitted for an elective bariatric surgery, Roux-en-y gastric bypass. His post-operative course was complicated by early ventilator-associated-pneumonia, septic shock, acute renal failure and right middle cerebral artery stroke. He had a lengthy intensive care unit (ICU) stay and required hemodialysis and tracheostomy. He was transferred to a long-term acute care hospital (LTACH) after 29 days in the acute care hospital ICU.

In the LTACH, the patient’s condition gradually improved with return of his kidney function and successful liberation from mechanical ventilation. The patient, however, continued to remain bedridden due to extensive left sided weakness and severe deconditioning. During this hospitalization, he also developed buttocks decubitus ulcers that was managed conservatively. In the 9^th^ week of his illness he developed a new fever of 38.2°C. He had no respiratory symptoms, his tracheostomy tube was already removed and his chest X-ray was negative. He had no gastrointestinal symptoms and his abdominal exam, including the surgical site, was unremarkable. He had a foley catheter but his urinalysis and urine culture were negative. He had a single peripheral intravenous catheter without any surrounding redness or tenderness. Two sets of blood cultures were obtained but were negative.

**Figure 1:**
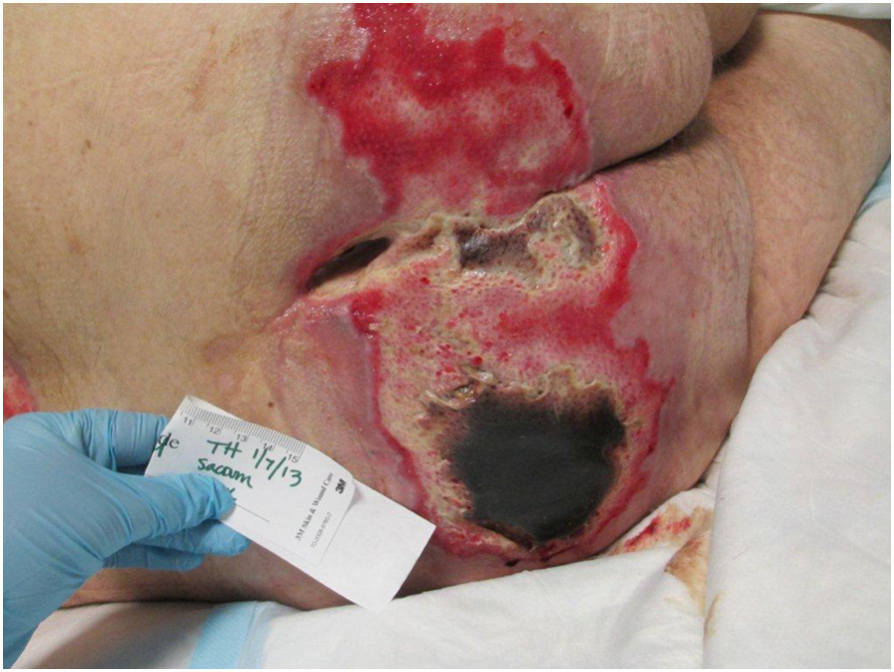
The patient’s unstageable decubitus ulcers with a black eschar covering the left buttocks ulcer.

**Figure 2:**
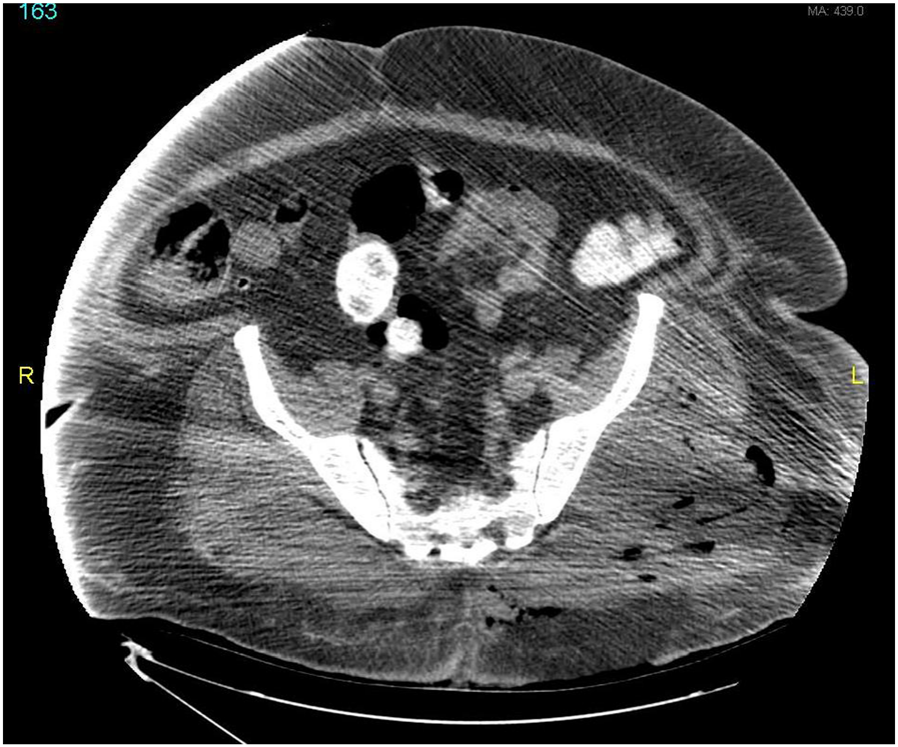
Computed tomography cross section of the pelvis showing a skin defect corresponding to the eschar that was removed with underlying fat stranding, inflamed muscles and gas pockets throughout tissue planes.

Despite several days of broad-spectrum antibiotic coverage with vancomycin and pipracillin/tazobactam he continued to have low-grade fevers. On examination, the left buttock decubitus ulcer had a black eschar with foul smell (Figure 1). Surgical consultation was placed; the patient underwent computed tomography scan of the pelvis (Figure 2) and bedside limited debridement with collection of a swab culture (BBL CultureSwab collection and transport system, Becton, Dickinson and company, Sparks, MD, USA) from the base of the wound after removing the eschar. Shortly afterwards, he had a fever of 40.3°C and he became confused and hypotensive. The patient was transferred back to the ICU of the acute care hospital with severe sepsis. His white blood cell count was 17.3 × 10^9^/L with 20% bands and his creatinine increased to 1.8 mg/dl. Two sets of blood culture (VersaTrek, TREK Diagnostic Systems, Cleveland, OH, USA) were collected and his antibiotics were broadened to linezolid and meropenem and he received aggressive intravenous fluid resuscitation. He was taken back to the operating room for several rounds of debridement that involved resection of significant amounts of skin and soft tissues of both buttocks with removal of the devitalized left sided gluteal muscles.

**Figure 3:**
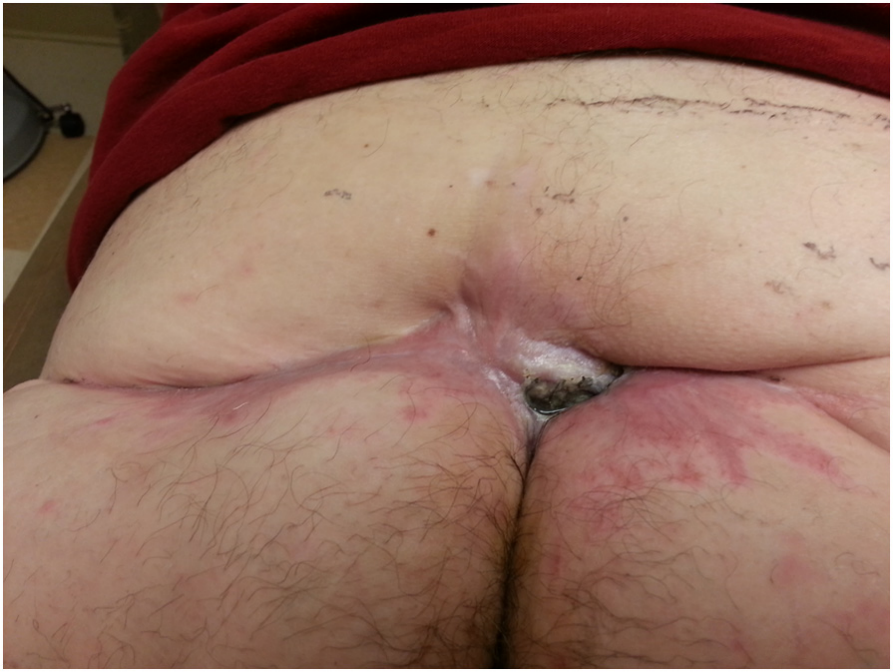
The patient’s healing decubitus ulcer 9 months into treatment.

The anaerobic bottles in both blood culture sets grew *Prevotella loescheii* and the surgical sample grew extended-spectrum beta-lactamase (ESBL) producing *Escherichia coli*, ampicillin-susceptible *Enterococcus faecalis* and *P.loescheii* ***.*** Bacterial identification was performed by Microscan WalkAway plus system (Siemens, Germany). No susceptibility testing was performed on *P. loescheii* and antibiotics with well-reported activity against anaerobes, carbapenems, were used. The patient’s sepsis gradually improved. His antibiotics were subsequently de-escalated to ertapenem and he finished a total of 3 weeks of therapy after the last debridement session. His left buttock wound continued to heal slowly over the course of several months (Figure 3).

## Discussion

We presented a case of bactermic, infected decubitus ulcer in a young, albeit, debilitated, chronically ill patient. The role of *P. loescheii* in this mixed-infection is indisputable given the fact that it grew in both wound and blood cultures [2]. We speculate that perineal and colonic flora contaminated and subsequently infected the above-described decubitus ulcer. The oxygen deficient environment of the decubitus wound coupled with extensive tissue necrosis created the perfect environment for this anaerobic bacterium to grow and thrive. Bedside surgical debridement allowed *P. loescheii* to invade the blood stream causing bacteremia and severe sepsis.

*Prevotella* spp. are non-motile, Gram-negative anaerobic bacilli. They are usually isolated from the mucosal surfaces of the mouth, colon and vagina in otherwise healthy individuals [5-8]. This anaerobe is considered an opportunistic pathogen [7]. To date, more than 50 species of *Prevotella* have been identified [9]. They have been implicated in the pathogenesis of periodontal infections with potential spread to adjacent structures [4,10-13]. Recently, however, there have been a number of cases in which *Prevotella* spp. caused infections at a distance from their primary source [14,15].

Like all anaerobes, isolation of *Prevotella* spp. is difficult and requires appropriate methods for sample collection, transportation and culture. *In vitro* susceptibility testing of anaerobes is not performed routinely in many hospitals’ clinical labs, including our hospital’s microbiology lab. Hence, treatment of these infections is largely empiric and relies on reported susceptibilities from large medical centers and reference labs [16]. Of note, resistance among anaerobes has been rising; a number of studies have demonstrated increasing resistance among *Prevotella* spp. against β-lactam antibiotics and some have shown emergence of partial resistance to metronidazole as well [12,17-21].

Literature search revealed five reported cases of *P. loescheii* infection outside the oral cavity [12,14,15]. The salient features of cases reported in English language including the one at hand are summarized in Table 1. There was a case of poly-microbial brain abscess in a child reported in Japanese and a case of mixed infection in a foot ulcer reported in French from Djibouti [22]. None of these 5 cases were associated with *P. loescheii* bacteremia at the time of diagnosis. Upon reviewing the above cases the following was noticeable:

1. Patients’ ages varied widely but they were all males.

2. Large intestine was the source of infection in our patient, while the oral-nasal cavity was the source of bacteria in other cases.

3. The nosocomial case presented here was in contrast to the others, which were all community-acquired.

4. The presented case was polymicrobial, while the others were mono-microbial.

5. None of the reported cases died but they all required a lengthy course of antibiotic therapy.

**Table 1:** **Important features of reported *P. loescheii *** **infection cases**

**Patient characteristics**	**Co-morbidity**	**Infection**	**Cultured sample**	**Susceptibility testing**	**Treatment**	**Ref. no.**
42 yo M	Decubitus ulcer in an obese bedridden patient	Bacteremic skin and soft tissue infection	Blood and a surgical sample	ND	Ertapenam for 3 weeks	Current case
75 yo M	Dental extraction in a patient with osteoarthritis	Septic arthritis	Synovial fluid	Susceptible to PCN, BL/BL inhibitor, metronidazole	Amoxacillin-clavulante for 4 weeks	[15]
62 yo M	Recurrent sinusitis	Brain subdural abscess	Pus	Susceptible to PCN, BL/BL inhibitor, clindamycin, chloramphenicol.	Chloramphenicol and clindamycin for 5 weeks	[12]
				Resistant to metronidazole.		
20 yo M	Dental procedures in a patient with R. hip replacement	R. hip prosthetic joint infection	Surgically obtained bone samples	ND	Cefuroxime and fosfomycin X 6 weeks	[14]

**Abbreviations:***yo* year-old, *M* male, *ND* not done, *PCN* penicillin, *BL*/*BL* Beta*-*lactam/Beta-lactamase, *R* right, *ref. No* reference number.

## Conclusion

Our case, underscores the potential increase in virulence among opportunistic pathogens like *P. loescheii,* especially in the ever-growing population of medically compromised patients ***.*** With changing antimicrobial resistance described over the last three decades, further work is needed to identify these anaerobes’ mechanism of transmission, spectrum of disease and best antimicrobial therapy.

## Consent

Written informed consent was obtained from the patient for publication of this case report and any accompanying images. A copy of the written consent is available for review by the Editor of this journal.

## Competing interests

The authors declared that they have no competing interests.

## Authors’ contributions

FAK and MN diagnosed and treated the patient. MM and NAJ reviewed the data and the literature and they wrote the manuscript’s initial draft. FAK and MN edited the manuscript. All authors read and approved the manuscript.

## Pre-publication history

The pre-publication history for this paper can be accessed here:

http://www.biomedcentral.com/1471-2334/14/162/prepub
